# The *ACE* I/D polymorphism does not explain heterogeneity of natural course and response to enzyme replacement therapy in Pompe disease

**DOI:** 10.1371/journal.pone.0208854

**Published:** 2018-12-07

**Authors:** Esther Kuperus, Jan C. van der Meijden, Stijn L. M. in ’t Groen, Marian A. Kroos, Marianne Hoogeveen-Westerveld, Dimitris Rizopoulos, Monica Yasmin Nino Martinez, Michelle E. Kruijshaar, Pieter A. van Doorn, Nadine A. M. E. van der Beek, Ans T. van der Ploeg, W. W. M. Pim Pijnappel

**Affiliations:** 1 Center for Lysosomal and Metabolic Diseases, Erasmus MC University Medical Center, Rotterdam, the Netherlands; 2 Department of Neurology, Erasmus MC University Medical Center, Rotterdam, the Netherlands; 3 Department of Pediatrics, Erasmus MC University Medical Center, Rotterdam, the Netherlands; 4 Department of Clinical Genetics, Erasmus MC University Medical Center, Rotterdam, the Netherlands; 5 Department of Biostatistics, Erasmus MC University Medical Center, Rotterdam, the Netherlands; Tor Vergata University, ITALY

## Abstract

The majority of children and adults with Pompe disease in the population of European descent carry the leaky splicing *GAA* variant c.-32-13T>G (IVS1) in combination with a fully deleterious *GAA* variant on the second allele. The phenotypic spectrum of this patient group is exceptionally broad, with symptom onset ranging from early infancy to late adulthood. In addition, the response to enzyme replacement therapy (ERT) varies between patients. The insertion/deletion (I/D) polymorphism of the angiotensin I-converting enzyme (*ACE*) has been suggested to be a modifier of disease onset and/or response to ERT. Here, we have investigated the effect of the *ACE* I/D polymorphism in a relatively large cohort of 131 children and adults with Pompe disease, of whom 112 were followed during treatment with ERT for 5 years. We assessed the use of wheelchair and mechanical ventilation, muscle strength assessed via manual muscle testing and hand-held dynamometry (HHD), distance walked on the six-minute walk test (6MWT), forced vital capacity (FVC) in sitting and supine position and daily-life activities assessed by R-PAct. Cross sectional analysis at first visit showed no differences between the genotypes with respect to age at first symptoms, diagnosis, wheelchair use, or ventilator use. Also response to ERT over 5 years assessed by linear mixed model analyses showed no significant differences between *ACE* groups for any of the outcome measures. The patient cohort contained 24 families with 54 siblings. Differences in ACE genotype could neither explain inter nor intra familial differences. We conclude that the *ACE* I/D polymorphism does not explain the large variation in disease severity and response to ERT observed among Pompe patients with the same c.-32-13T>G *GAA* variant.

## Introduction

Pompe disease (OMIM 232300) is a metabolic myopathy caused by disease-associated variants in the acid α-glucosidase *(GAA)* gene (OMIM 606800). This results in deficiency of the lysosomal enzyme GAA, leading to an impaired breakdown of glycogen [1]. Clinically, a broad disease spectrum can be observed, ranging from a rapidly progressive classic infantile phenotype to a slower progressing disease course in children and adults. Classic infantile patients present shortly after birth with hypertrophic cardiomyopathy and generalized muscle weakness. Without treatment these patients die within the first year of life due to cardiorespiratory insufficiency [2, 3]. Children and adults present with a slower progressive limb girdle muscle weakness, while cardiac involvement is rare. Most of these patients become wheelchair and ventilator dependent. Survival is reduced compared to the general population [4–6].

The majority of children and adults of European descent with Pompe disease carry the common c.-32-13T>G (IVS1) variant on one *GAA* allele. The IVS1 variant causes aberrant *GAA* pre-mRNA splicing by inducing partial or complete skipping of *GAA* exon 2. A small percentage (10–15%) of leaky wild type splicing occurs [7–12]. The second *GAA* allele in children and adults with Pompe disease is in many cases a ‘null’ allele, which is defined as an allele that does not generate any detectable *GAA* enzymatic activity, for example an allele that carries the frequent variant c.525delT [13]. Interestingly, the natural disease course in patients with the IVS1 variant shows an exceptionally broad spectrum: disease onset can vary from early infancy to late adulthood and symptoms can vary. Such differences are even observed among patients with the same IVS1/c.525delT *GAA* genotype [14–17]. The variation between siblings is less broad, suggesting that disease progression can be modified by genetic background factors [17].

Since 2006, enzyme replacement therapy (ERT) with recombinant human *GAA* (rhGAA), is available for Pompe disease. In children and adults with Pompe disease ERT has shown to improve muscle function and strength and to stabilize pulmonary function. However, individual responses can vary considerably [18], which is highlighted in our recent study on the clinical response to ERT during 5 year follow up [19]. This is observed irrespective of formation of anti-rhGAA antibodies, suggesting that other factors exist that can modify the response to ERT in this patient group [19–21].

It has been suggested that a polymorphism in the angiotensin converting enzyme (*ACE*) gene—the insertion (I) or deletion (D) of an alu repeat in intron 16—may affect phenotypic variation and response to ERT in patients with Pompe patients [22–25]. So far, four studies were performed, but the outcomes of the studies were different [22–25]. The DD genotype was associated with an earlier disease onset in some studies [22, 23], but not in another [25], and it was associated with less favorable response to ERT with regard to muscle mass in one study [24], and with regard to FVC and 6MWT in another study [25]. The different outcomes may be explained by small group sizes, an overrepresentation of adult patients, and/or a short follow up of maximum 2 years. This was reason to perform the current nationwide study in a group of 131 children and adults representing the full phenotypic spectrum of patients with the c.-32-13T>G/null gene type. The aim was to further explore the potential influential effect of the *ACE* polymorphism on age of disease onset, disease severity and outcome of patients when treated with ERT by using a relatively large patient cohort and a longer follow up of 5 years of ERT.

## Materials and methods

### Patients and study design

This study was part of an ongoing single-center prospective, open-label study, in which all Dutch children and adults with a confirmed diagnosis of Pompe disease—by enzyme analysis in leucocytes or fibroblasts, and by DNA analysis—participated [15, 26–28]. Only patients that carried the c.-32-13 T>G (IVS1) *GAA* variant on one allele and a fully deleterious (“null”) *GAA* variant on the other allele were included in this study. Information was collected with respect to onset of symptoms, age of diagnosis and wheelchair- or ventilator dependency. Clinical assessments were performed with 3–6 months intervals. Daily life activities were assessed via the Rasch-built Pompe-specific Activity (R-PAct) scale [29]. Data were collected from January 1, 1999 through January 1, 2016. All data available during this period were used in this analysis. The study was conducted according to the Declaration of Helsinki, the Medical Ethical Committee at Erasmus MC University Medical Center approved the study protocol, and all patients, or their parents or legal guardians, provided written informed consent.

### ACE polymorphism

*ACE* genotyping was performed based on the methods described by Al-Awadhi et al [30]. In short, genomic DNA from blood or fibroblasts was used in a first PCR flanking the alu insertion in intron 16 using the following primers: fw1: 5’-CTGGAGACCACTCCCATCCTTTCT-3’ and rev1: 5’-GATGTGGCCATCACATTCGTCAGAT-3’. PCR was performed using FastStart PCR (Roche) in which 6% DMSO was included in the reaction mixture. Reactions were run on a Bio-rad C1000 Touch Thermal Cycler. When no I allele was found, a second PCR was performed using an alu insertion-specific internal primer fw2: 5’-TGGGATTACAGGCGTGATACAG-3’, which was used in a PCR with the same primer rev1 as above. Positive, negative, and blank controls for the *ACE* genotype were included in all analyses.

### Clinical outcome measures

The use of a wheelchair and mechanical ventilation was registered at each visit. Skeletal muscle strength was assessed using the Medical Research Council (MRC) grading scale and hand-held dynamometry (HHD; Cytec dynamometer, Groningen, The Netherlands) [31–33]. The following muscle groups were tested for either method: neck flexors, shoulder abductors, elbow flexors, elbow extensors, hip flexors, hip abductors, knee flexors, and knee extensors. Additionally, an MRC grade was determined for the hip extensors and hip adductors. This was expressed as percentage of the maximum possible score for MRC sum scores. HHD sum scores were expressed as percentage of the median strength of healthy males and females. Sum scores were calculated if no more than two muscle groups were missing. Muscle function was assessed using the Quick Motor Function Test (QMFT), consisting of 16 motor skills related to daily life [34]. Muscle endurance was assessed using the six-minute walk test (6MWT) in which the distance walked in 6 minutes was recorded [35]. Forced vital capacity (FVC) was measured in upright and supine positions. Results were expressed as percentage of the predicted FVC [36]. The R-PAct scale was used to assess patients’ self-reported ability to perform daily life activities. A score was calculated as described [29], only when all items had been answered. Only adult patients performed this test.

### Statistical analysis

Differences in characteristics between the three different *ACE* genotypes (II, DD and ID) at first visit were calculated as follows. First we tested if any of the *ACE* groups differed from the others using the Kruskal-Wallis test for numerical data and the chi-square test (2x3) for categorical data. When significant, the Mann-Whitney and chi-square tests (2x2) were used to identify which of the groups (II vs ID, II vs DD or ID vs DD) differed. We corrected for multiple testing using the Holm method [37].

Longitudinal analyses of the effects of ERT were performed using linear mixed effect models to account for repeated measurements per patient as described [19]. Models were fitted for each outcome measure using the nlme package of the statistical program R (version 3.2.5) [38, 39]. Time was expressed as years after start of ERT. To account for potential non-linear profiles we used natural cubic splines in the fixed-effects and random-effects parts of the model. In the specification of the splines, boundary knots were placed at 0 (i.e. start of ERT) and 5 years, and internal knots were placed at 1 and 3.5 years. For the random-effects part of the model an unstructured covariance matrix was used. Likelihood-ratio-tests were used to asses if there was an interaction between time and *ACE* polymorphism; e.g. if outcome measures progressed differently during treatment for each *ACE* polymorphism or if the different polymorphisms had different intercepts; e.g. overall disease severity. Obtained p-values were corrected for multiple testing using the Holm method [37]. Plots for the group means were generated for the different outcome measures during 5 years of ERT as described [19]. Siblings from various families were identified. For each sibling the age at first symptoms, diagnosis, the start of ERT, the start of wheelchair/ventilator use, and their last follow-up or death were plotted to study if different *ACE* genotypes explained variation within families. Siblings were classified as having varying outcomes when an event occurred in one sibling and did not occur within 10 years in the other sibling.

### Power analysis

We conducted a post hoc power analysis based on our cross-sectional data using the outcome measure age of onset, as this is highly variable in patients with the same IVS-1/null variants. We assumed that the ACE genotype would fully explain all phenotypical variation between patients. In this scenario, the medians of the three ACE genotype groups (II, ID, and DD) are the same as the 25^th^, 50^th^ and 75^th^ percentiles of the age of onset of the overall population. We calculated how many subjects are needed to detect a difference between ACE groups at a power of >0.8 by simulating a thousand ‘sample populations’ with the given number of patients, and performing a Kruskal-Wallis test.

## Results

### Patients

A total of 146 Dutch patients with the IVS1/”null” genotype were known in our center at data closure. DNA was available for 131 patients to determine the *ACE* genotype (Table 1), of whom 112 had started with ERT. The characteristics of the total patient group (n = 131) were as follows. Gender was evenly distributed (50% females) in the total group and the three different *ACE* groups (II, DD and ID). Most patients (86%) started ERT at adulthood (>18 years of age). Median ages (in years, with ranges indicated in brackets) at symptom onset, diagnosis, first visit, and start of ERT were 31 (0–62); 38 (0–72); 46 (0–75); and 49 (1–76) years, respectively. Further testing of the age range at start of ERT revealed that these did not differ between the ACE groups. At first visit, 31% of patients used a wheelchair, while the median age at which patients started to use a wheelchair was 49 (11–76) years. For usage of a ventilator, these numbers were 22% and 52 years of age (6–72). The age ranges for all parameters listed above were broad, highlighting the heterogeneity of disease onset and progression of Pompe patients with the IVS1 variant.

**Table 1:** Patient characteristics at first visit.

	Total (n = 131/112^#^)	ACE polymorphism			p-value
		II (n = 32; 24%)	DD (n = 41; 31%)	ID (n = 58)	
Gender, No. of patients (%)					
- Male	65 (50%)	15 (47%)	20 (49%)	30 (52%)	
- Female	66 (50%)	17 (53%)	21 (51%)	28 (48%)	n.s.
Start ERT during childhood (<18y), n (%)^#^					
- Yes	13 (12%)	6 (22%)	2 (6%)	5 (10%)	
- No	99 (88%)	20 (77%)	32 (94%)	46 (90%)	n.s.
Median age (range), at:					n.s.
Onset of symptoms	31 (0–62)	28 (0–54)	33 (4–61)	30 (0–62)	n.s.
Diagnosis	38 (0–72)	35 (0–69)	42 (0–67)	38 (0–72)	n.s.
First visit	46 (0–75)	41 (2–69)	47 (6–71)	47 (0–75)	n.s.
Start ERT^#^^§^	49 (1–76)	42 (1–68)	50 (14–73)	50 (1–76)	n.s.
Wheelchair use at first visit, n (%)					
- No	91 (69)	22 (69)	23 (56)	46 (79)	
- Yes	40 (31)	10 (31)	18 (44)	12 (21)	n.s.*
Wheelchair age, median (range)	49 (11–76)	43 (11–60)	51 (24–64)	55 (33–76)	n.s.
Ventilation use at first visit, n (%)					
- No	102 (78)	28 (88)	31 (76)	43 (74)	
- Yes	29 (22)	4 (12)	10 (24)	15 (26)	n.s.
Ventilation age, median (range)	52 (6–72)	53 (33–61)	48 (6–72)	51 (13–69)	n.s.

*Null hypothesis II = ID = DD rejected at the p<0.05 level. Post-hoc testing (II vs ID, II vs DD or ID vs DD) did not show significant differences between the ACE groups.

^#^All parameters were tested for 131 patients, except for start ERT during childhood and median age at start ERT for which a total of 112 patients were analyzed.

^§^ age ranges were n.s.

### Effect of *ACE* I/D polymorphism at first visit: Cross sectional analysis

The *ACE* polymorphism genotype was normally distributed within the total patient group (II: 24%; ID: 44%; DD: 31%) (Table 1). Outcomes were compared between the three groups in a cross sectional analysis at first visit (Table 2A) and at start of ERT (Table 2B). This showed that none of the parameters were different between the groups. The use of a wheelchair at first visit was initially found to be different with a p-value of 0.047 with the lowest number of wheelchair users in the DD group, but post-hoc testing showed that this was not significant. Slightly more II patients started ERT during childhood, but the differences between *ACE* groups were also not significant.

**Table 2:** Cross-sectional evaluation of muscle force, function and lung function at first visit (A) and start of ERT (B).

	Total (n = 131)	**A. Outcomes at first visit**			
		II (n = 32)	DD (n = 41)	ID (n = 58)	p-value
HHD; % of maximal score (range)	73 (32–99)	75 (33–95)	73 (3–97)	73 (32–99)	n.s.
MRC; % of maximal score (range)	83 (33–100)	86 (55–100)	81 (51–100)	84 (33–100)	n.s.
QMFT; % of maximal score (range)	67 (14–100)	80 (22–100)	64 (14–100)	65 (17–100)	n.s.
R-PAct; R-Pact score (range)	54 (7–100)	58 (7–100)	50 (7–94)	55 (17–83)	n.s.
6MWT; meters walked (range)	436 (48–650)	455 (75–645)	347 (82–544)	448 (48–650)	n.s.
FVC sitting; % of predicted (range)	73 (10–117)	84 (10–117)	73 (15–107)	72 (15–107)	n.s.
FVC supine; % of predicted (range)	61 (17–107)	71 (18–107)	68 (24–104)	48 (17–105)	n.s.
	Total (n = 112)	**B. Outcomes at start ERT**			
		II (n = 26)*	DD (n = 35)*	ID (n = 51)*	p-value
HHD; % of maximal score (range)	70 (26–100)	70 (33–100)	66 (26–95)	73 (26–95)	n.s.
MRC; % of maximal score (range)	82 (47–99)	84 (55–99)	79 (53–96)	82 (47–99)	n.s.
QMFT; % of maximal score (range)	74 (28–100)	57 (14–94)	57 (14–94)	63 (13–97)	n.s.
R-PAct; R-Pact score (range)	52 (7–86)	59 (7–86)	44 (7–75)	56 (17–83)	n.s.
6MWT; meters walked (range)	417 (41–650)	436 (75–645)	353 (82–626)	435 (41–650)	n.s.
FVC sitting; % of predicted (range)	57 (15–111)	84 (41–111)	57 (15–110)	62 (18–105)	n.s.
FVC supine; % of predicted (range)	52 (16–111)	65 (16–111)	58 (24–98)	44 (17–96)	n.s.

Abbreviations: ERT = enzyme replacement therapy; HHD = handheld dynamometry; MRC = Medical Research Council; QMFT = Quick Motor Function Test; R-PAct = Rasch-Built Pompe-Specific Activity scale; 6MWT = 6-minute walk test; FVC = forced vital capacity percentage predicted; n.s. = not significant.

*The age ranges of patients that had started with ERT were 1–68 for the II group, 14–73 for the DD group and 1–76 for the ID group, and these were not significantly different between the ACE genotype groups.

### Effect of *ACE* I/D polymorphism on response to enzyme replacement therapy

**Fig 1:**
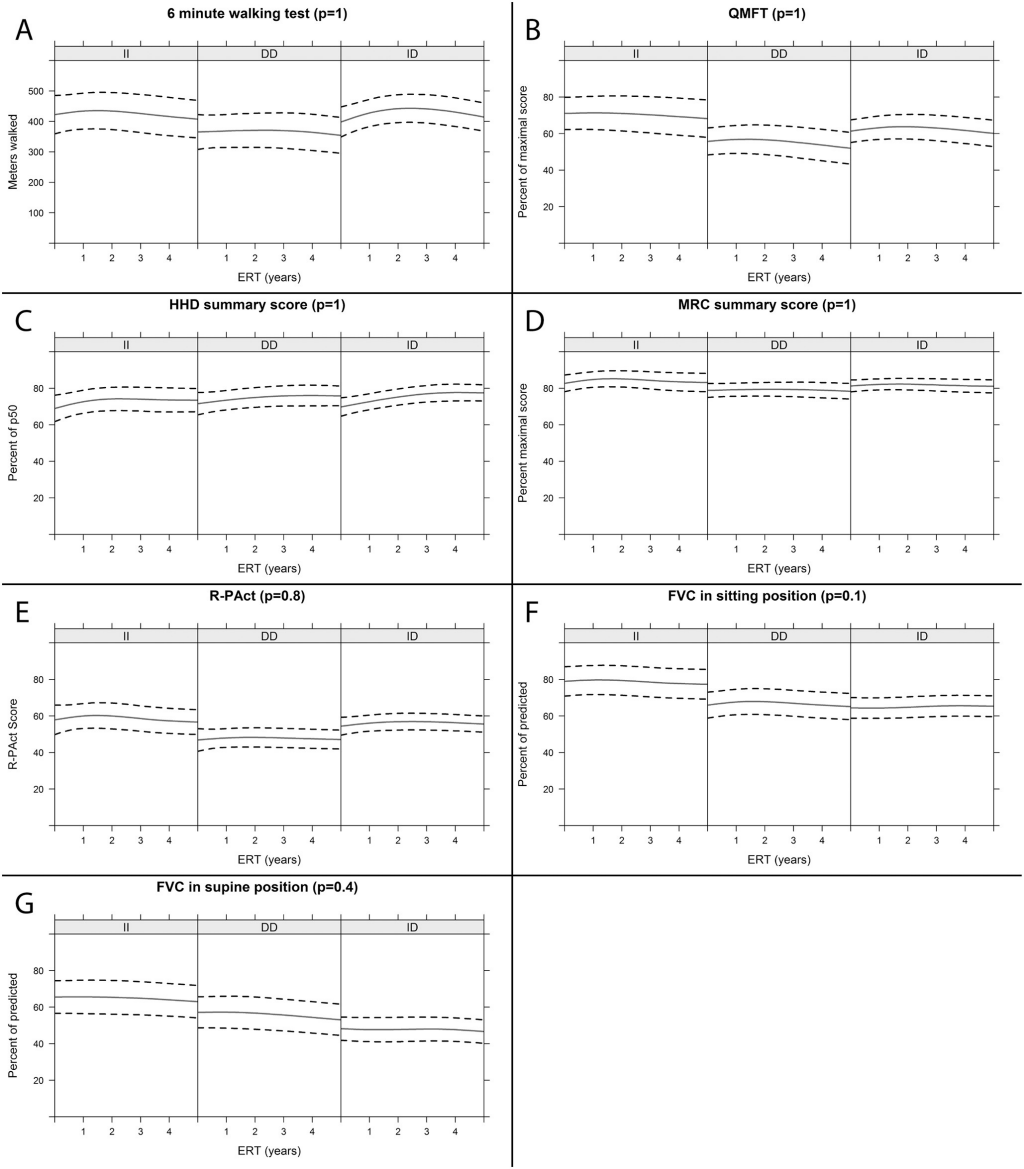
Predicted group means for outcome measures over 5 years of ERT treatment. Group means (continuous line) of the outcome measures and 95% prediction interval (area between the dotted lines) obtained for the II, ID and DD genotypes using linear mixed models. P values are indicated in the titles above the graphs.

A total of 112 patients within the cohort received ERT and were included in longitudinal analysis of 5 years follow-up (Fig 1). We followed the response to ERT by assessment of muscle strength (MRC sumscore, HHD), muscle function (6 minute walking test, QMFT), respiratory function (FVC in sitting and supine positions), and daily life activities (R-Pact scale). The results were analyzed using linear mixed effects models. For some parameters, a difference was observed at the start of ERT between the II and the DD genotype groups, notably for R-Pact scale, QMFT, and FVC in sitting position, with a more favorable value for the II group. However, following multiple testing correction, these differences were not significant. The responses to ERT (i.e. the slopes of the curves during ERT treatment) were not different between the ACE genotype groups for all parameters tested. Individual responses to ERT varied within the ACE genotype groups. We found both good responders and non-responders to ERT in all three ACE genotype groups.

### *ACE* polymorphisms within families

**Fig 2:**
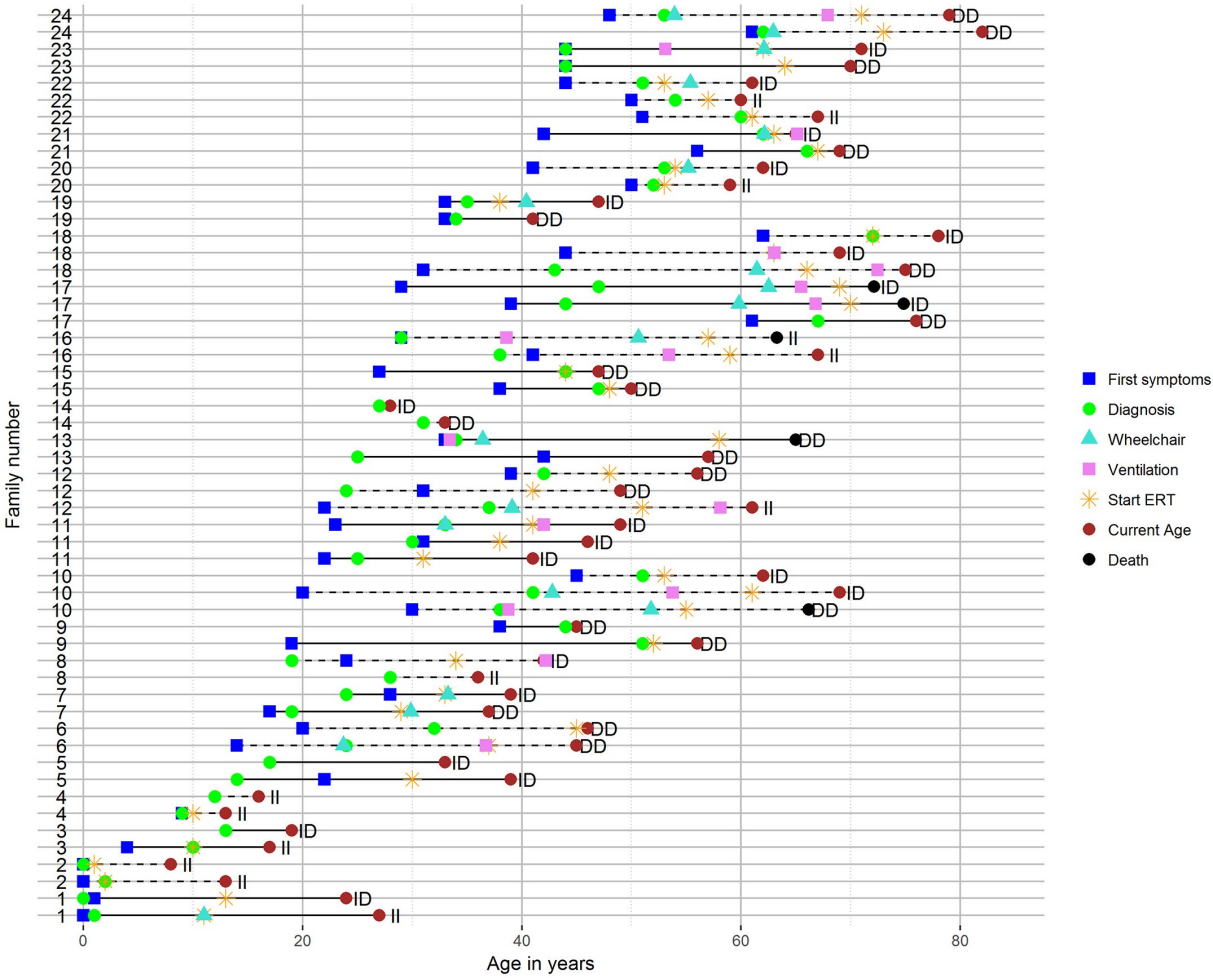
*ACE* polymorphisms and clinical parameters in family members with Pompe disease. Each line represents one patient. Families are numbered and visualized using alternating straight and dotted lines. The *ACE* genotype of individual patients is indicated at the right side of each patient’s line. Onset of clinical events is plotted on the X axis and indicated with the symbols indicated on the right.

Our cohort of patients included 54 siblings from 24 different families (2–3 siblings per family). Age at first symptoms, diagnosis, start of wheelchair use, ventilator use, ERT, death (if applicable), and *ACE* genotype are shown for each patient in Fig 2. Families are ordered by the onset of disease of the youngest family member. In 14 of the 24 families, siblings had discordant *ACE* polymorphisms, while 10 had the same *ACE* polymorphism. We found siblings with discordant *ACE* genotypes but with similar disease courses (8 siblings from 4 families; families 14, 19, 20 and 22), while we also found siblings with the same *ACE* genotype but very different disease courses (21 siblings from 10 families; families 5, 6, 9, 10, 11, 13, 15, 16, 18 and 24). We conclude that no clear influence of the *ACE* genotype on onset of disease symptoms can be detected within these families.

## Discussion

Pompe patients with the IVS1 variant represent the largest patient group of European descent with childhood or adult disease onset. These patients have a particularly large variation in phenotype, with symptom onset ranging from 0 to 62 years of age [5, 15, 40]. Pompe patients in general have a variable response to ERT, ranging from good to moderate to non-responders [19, 21, 41]. Here, we investigated the potential contribution of the *ACE* I/D genotype as a modifier of Pompe disease and response to ERT. Our results indicate that such contribution, if it exists, is too small to explain the phenotypic heterogeneity in this patient group. This applies both to differences in disease severity and to the response to ERT. Analysis of siblings with the same IVS1 genotype also did not point to a contribution of *ACE* genotype to clinical variation within families. These results are discussed in the light of other reports and with respect to the statistical power needed to identify a genetic modifier for Pompe disease.

A priori, at least two categories of genetic factors can be envisioned as potential modifiers of the disease course in Pompe disease. The first is a modifier of splicing, because the IVS1 variant is a splicing variant. For example, in theory it is possible that polymorphisms in splicing factors affect the amount of leaky wild type splicing by the IVS1 variant. If such polymorphism would exist, it would likely be present in a general splicing factor that is shared between fibroblasts and skeletal muscle cells, because these two cell types show similar aberrant splicing patterns [42]. Another category might be a genetic factor that modifies skeletal muscle function, as skeletal muscle is affected in Pompe disease. The *ACE* I/D genotype falls in this category, as it has been associated with performance of top athletes. Depending on the type of sport and its requirement for either endurance or strength, groups of top athletes are either enriched in the I or the D *ACE* alleles, respectively [43–49]. Considering the exceptionally broad clinical phenotype of patients with the IVS1 variant, a strong modifier is expected to explain the large variation in disease severity. Alternatively, modulation of the phenotype may occur via a combination of genetic factors with small effects that, when combined, have a strong effect on muscle function. In this scenario, the *ACE* polymorphism might be one of multiple factors that, when combined, affect the clinical phenotype.

The first study on the effect of the *ACE* I/D genotype on the natural course of Pompe patients was published in 2010. This study included 38 patients, 36 of whom contained the IVS1 *GAA* variant [22]. Sixteen parameters were tested in cross sectional analyses. The results suggested significant differences for some parameters, notably an association between the DD genotype and a worse Walton score (which scores muscle function), an earlier disease onset, more muscle pain, and higher CK levels were found. In 2014, the initial study was extended to 85 patients that contained the IVS1 variant [23]. Associations were found between the DD genotype and pain, but in contrast to the previous study, no associations between *ACE* genotype and Walton score, CK levels or other clinical parameters were found in cross sectional analyses. Another study was published in 2016, in which 58 patients that had previously been included in the treatment arm of the late onset placebo controlled multicenter enzyme replacement study (LOTS study) were analyzed [25]. In this study, very mildly affected and severely affected patients were excluded. This showed no association between *ACE* genotype and any parameter including onset of first symptoms, disease duration, 6MWT, or FVC rating disease severity. In the present study, no inclusion criteria were applied other than the presence of the IVS1 variant combined with a null allele and a confirmed diagnosis of Pompe disease. Given the conflicting results published so far, we aimed to test the effect of the *ACE* genotype in a relatively large patient cohort of 131 patients including both children and adults of various ages and different disease severities. This revealed no significant effects of the *ACE* genotype on any of the parameters tested. It should be noted that initial statistical analysis suggested some significant differences between different *ACE* genotype groups, but that these turned into non-significant p-values after multiple testing correction, a method that has not always been applied in previous studies. When we only included adult patients, there were no significant effects of the ACE genotype on any parameter tested (data not shown). Taken together, the initial idea that the *ACE* DD genotype may be associated with faster symptom onset and more severe muscle symptoms could not be confirmed.

Two previous studies have investigated the influence of the ACE I/D genotype on the response to treatment with ERT. In a study on 16 patients with the IVS1 allele that were treated for > 2 years with ERT, the DD genotype (n = 3) was associated with reduced muscle mass over the course of treatment, while no associations were found for muscle strength, FVC, or 6MWT [24]. In the previously mentioned LOTS study, in which 58 moderately affected patients were treated with ERT for 78 weeks, the DD genotype (n = 17) was associated with a poorer response to ERT of FVC in sitting position [25]. One other parameter was tested, namely the 6MWT, and this showed a better response in patients with the ID genotype and a trend toward a better response in patients with the II genotype compared to the DD genotype. Other parameters for muscle strength and function were not reported in this study. The overall conclusion from the LOTS study was therefore that the II genotype may be associated with a better response to ERT. In the present study, 112 patients were treated with ERT with a follow-up of 5 years after start of ERT, and severely affected patients were also included. Patients with the DD genotype in general did worse compared to the II genotype for several parameters, including 6MWT, QMFT, QMFT, FVC, and R-Pact scale. However, the effects were small and not statistically significant. Altogether, the data available to date do not support the idea that the *ACE* genotype can explain the heterogeneous response to ERT in juvenile and adult Pompe patients.

The question arises whether we would have been able to detect an effect of the *ACE* genotype in our patient cohort, given the number of patients tested in relation to the heterogeneity of clinical outcome. To address this, we performed a post-hoc power analysis. We performed the calculation for the hypothetical situation in which the *ACE* genotype is the only modifying factor that is responsible for phenotypic variation in patients with the IVS1 variant. Although we realize that this may not be the case, the calculation has been performed in this way to give a sense of the number of patients required. As a primary outcome measure, we used symptom onset, because this outcome measure shows a documented large variation among IVS1 patients [17]. In our patient cohort, we calculated that 12 patients would be sufficient to demonstrate a significant effect on natural course (power = 0.96). In this study, we included 131 patients with Pompe disease, arguing that we should have detected an effect of the *ACE* polymorphism in our cohort if it would exist. We conclude that a possible effect of the *ACE* I/D genotype on natural course or the clinical response to ERT, if any, is very small, and remains undetected in our patient cohort. The search for modifying factors that can explain phenotypic variation in Pompe disease should continue.

## References

[1] Hirschhorn R R A. Glycogen storage disease type II: acid alpha-glucosidase (acid maltase) deficiency In: Scriver CR B A, Sly WS, Valle D., editor. The Metabolic and Molecular Bases of Inherited Disease. 8th edition ed New York: McGraw-Hill; 2001 p. 3389–420.

[2] van den Hout HM, Hop W, van Diggelen OP, Smeitink JA, Smit GP, Poll-The BT, et al The natural course of infantile Pompe’s disease: 20 original cases compared with 133 cases from the literature. Pediatrics. 2003;112(2):332–40. Epub 2003/08/05. . 12897283 10.1542/peds.112.2.332

[3] Kishnani PS, Hwu WL, Mandel H, Nicolino M, Yong F, Corzo D, et al A retrospective, multinational, multicenter study on the natural history of infantile-onset Pompe disease. J Pediatr. 2006;148(5):671–6. Epub 2006/06/02. 10.1016/j.jpeds.2005.11.033 . 16737883 10.1016/j.jpeds.2005.11.033

[4] Beek NA, Hagemans ML, Reuser AJ, Hop WC, Ploeg AT, Doorn PA, et al Rate of disease progression during long-term follow-up of patients with late-onset Pompe disease. Neuromuscul Disord. 2009;19 10.1016/j.nmd.2008.11.007 19084399 10.1016/j.nmd.2008.11.007

[5] Beek NA, Vries JM, Hagemans ML, Hop WC, Kroos MA, Wokke JH, et al Clinical features and predictors for disease natural progression in adults with Pompe disease: a nationwide prospective observational study. Orphanet J Rare Dis. 2012;7. 10.1186/1750-1172-7-88 PMC3551719 23147228

[6] Laforet P, Nicolino M, Eymard PB, Puech JP, Caillaud C, Poenaru L, et al Juvenile and adult-onset acid maltase deficiency in France: genotype-phenotype correlation. Neurology. 2000;55(8):1122–8. Epub 2000/11/09. . 11071489 10.1212/wnl.55.8.1122

[7] Boerkoel CF, Exelbert R, Nicastri C, Nichols RC, Miller FW, Plotz PH, et al Leaky splicing mutation in the acid maltase gene is associated with delayed onset of glycogenosis type II. Am J Hum Genet. 1995;56(4):887–97. . 7717400 PMC1801206

[8] van der Wal E, Bergsma AJ, Pijnenburg JM, van der Ploeg AT, Pijnappel W. Antisense Oligonucleotides Promote Exon Inclusion and Correct the Common c.-32-13T>G GAA Splicing Variant in Pompe Disease. Mol Ther Nucleic Acids. 2017;7:90–100. Epub 2017/06/19. 10.1016/j.omtn.2017.03.001 . 28624228 10.1016/j.omtn.2017.03.001 PMC5415969

[9] Chen L, Bush SJ, Tovar-Corona JM, Castillo-Morales A, Urrutia AO. Correcting for differential transcript coverage reveals a strong relationship between alternative splicing and organism complexity. Mol Biol Evol. 2014;31(6):1402–13. Epub 2014/04/01. 10.1093/molbev/msu083 . 24682283 10.1093/molbev/msu083 PMC4032128

[10] Jangi M, Sharp PA. Building robust transcriptomes with master splicing factors. Cell. 2014;159(3):487–98. Epub 2014/11/25. 10.1016/j.cell.2014.09.054 . 25417102 10.1016/j.cell.2014.09.054 PMC4243530

[11] Lee Y, Rio DC. Mechanisms and Regulation of Alternative Pre-mRNA Splicing. Annu Rev Biochem. 2015;84:291–323. Epub 2015/03/19. 10.1146/annurev-biochem-060614-034316 . 25784052 10.1146/annurev-biochem-060614-034316 PMC4526142

[12] Bergsma AJ, Kroos M, Hoogeveen-Westerveld M, Halley D, van der Ploeg AT, Pijnappel WW. Identification and characterization of aberrant GAA pre-mRNA splicing in pompe disease using a generic approach. Hum Mutat. 2015;36(1):57–68. 10.1002/humu.22705 . 25243733 10.1002/humu.22705

[13] Kroos MA, Van der Kraan M, Van Diggelen OP, Kleijer WJ, Reuser AJ, Van den Boogaard MJ, et al Glycogen storage disease type II: frequency of three common mutant alleles and their associated clinical phenotypes studied in 121 patients. J Med Genet. 1995;32(10):836–7. Epub 1995/10/01. . 8558570 10.1136/jmg.32.10.836-a PMC1051720

[14] Kroos M, Hoogeveen-Westerveld M, van der Ploeg A, Reuser AJ. The genotype-phenotype correlation in Pompe disease. Am J Med Genet C Semin Med Genet. 2012;160C(1):59–68. Epub 2012/01/19. 10.1002/ajmg.c.31318 . 22253258 10.1002/ajmg.c.31318

[15] Kroos MA, Pomponio RJ, Hagemans ML, Keulemans JL, Phipps M, DeRiso M, et al Broad spectrum of Pompe disease in patients with the same c.-32-13T->G haplotype. Neurology. 2007;68(2):110–5. 10.1212/01.wnl.0000252798.25690.76 . 17210890 10.1212/01.wnl.0000252798.25690.76

[16] van Capelle CI, van der Meijden JC, van den Hout JMP, Jaeken J, Baethmann M, Voit T, et al Childhood Pompe disease: clinical spectrum and genotype in 31 patients. Orphanet Journal of Rare Diseases. 2016;11(1):65 10.1186/s13023-016-0442-y 27189384 10.1186/s13023-016-0442-y PMC4870771

[17] Wens SC, Gelder CM, Kruijshaar ME, Vries JM, Beek NA, Reuser AJ, et al Phenotypical variation within 22 families with Pompe disease. Orphanet J Rare Dis. 2013;8. 10.1186/1750-1172-8-182 PMC3843594 24245577

[18] van der Ploeg AT, Kruijshaar ME, Toscano A, Laforet P, Angelini C, Lachmann RH, et al European consensus for starting and stopping enzyme replacement therapy in adult patients with Pompe disease: a 10-year experience. Eur J Neurol. 2017;24(6):768–e31. Epub 2017/05/10. 10.1111/ene.13285 . 28477382 10.1111/ene.13285

[19] Kuperus E, Kruijshaar ME, Wens SCA, de Vries JM, Favejee MM, van der Meijden JC, et al Long-term benefit of enzyme replacement therapy in Pompe disease: A 5-year prospective study. Neurology. 2017;89(23):2365–73. 10.1212/WNL.0000000000004711 . 29117951 10.1212/WNL.0000000000004711

[20] de Vries JM, Kuperus E, Hoogeveen-Westerveld M, Kroos MA, Wens SC, Stok M, et al Pompe disease in adulthood: effects of antibody formation on enzyme replacement therapy. Genet Med. 2017;19(1):90–7. Epub 2016/07/01. 10.1038/gim.2016.70 . 27362911 10.1038/gim.2016.70

[21] van der Meijden JC, Kruijshaar ME, Harlaar L, Rizopoulos D, van der Beek N, van der Ploeg AT. Long-term follow-up of 17 patients with childhood Pompe disease treated with enzyme replacement therapy. J Inherit Metab Dis. 2018 10.1007/s10545-018-0166-3 . 29556838 10.1007/s10545-018-0166-3 PMC6326992

[22] de Filippi P, Ravaglia S, Bembi B, Costa A, Moglia A, Piccolo G, et al The angiotensin-converting enzyme insertion/deletion polymorphism modifies the clinical outcome in patients with Pompe disease. Genet Med. 2010;12(4):206–11. Epub 2010/03/24. 10.1097/GIM.0b013e3181d2900e . 20308911 10.1097/GIM.0b013e3181d2900e

[23] De Filippi P, Saeidi K, Ravaglia S, Dardis A, Angelini C, Mongini T, et al Genotype-phenotype correlation in Pompe disease, a step forward. Orphanet J Rare Dis. 2014;9:102 Epub 2014/08/12. 10.1186/s13023-014-0102-z . 25103075 10.1186/s13023-014-0102-z PMC4249737

[24] Ravaglia S, De Filippi P, Pichiecchio A, Ponzio M, Saeidi Garaghani K, Poloni GU, et al Can genes influencing muscle function affect the therapeutic response to enzyme replacement therapy (ERT) in late-onset type II glycogenosis? Mol Genet Metab. 2012;107(1–2):104–10. Epub 2012/06/19. 10.1016/j.ymgme.2012.05.016 . 22704482 10.1016/j.ymgme.2012.05.016

[25] Baek RC, Palmer R, Pomponio RJ, Lu Y, Ma X, McVie-Wylie AJ. The influence of a polymorphism in the gene encoding angiotensin converting enzyme (ACE) on treatment outcomes in late-onset Pompe patients receiving alglucosidase alfa. Mol Genet Metab Rep. 2016;8:48–50. Epub 2016/08/05. 10.1016/j.ymgmr.2016.07.005 27489778 10.1016/j.ymgmr.2016.07.005 PMC4961277

[26] van Diggelen OP, Oemardien LF, van der Beek NA, Kroos MA, Wind HK, Voznyi YV, et al Enzyme analysis for Pompe disease in leukocytes; superior results with natural substrate compared with artificial substrates. J Inherit Metab Dis. 2009;32(3):416–23. Epub 2009/04/24. 10.1007/s10545-009-1082-3 . 19387865 10.1007/s10545-009-1082-3

[27] Kroos M, Pomponio RJ, van Vliet L, Palmer RE, Phipps M, Van der Helm R, et al Update of the Pompe disease mutation database with 107 sequence variants and a format for severity rating. Hum Mutat. 2008;29(6):E13–26. Epub 2008/04/22. 10.1002/humu.20745 . 18425781 10.1002/humu.20745

[28] Okumiya T, Keulemans JL, Kroos MA, Van der Beek NM, Boer MA, Takeuchi H, et al A new diagnostic assay for glycogen storage disease type II in mixed leukocytes. Mol Genet Metab. 2006;88(1):22–8. Epub 2005/12/20. 10.1016/j.ymgme.2005.10.016 . 16359900 10.1016/j.ymgme.2005.10.016

[29] van der Beek NA, Hagemans ML, van der Ploeg AT, van Doorn PA, Merkies IS. The Rasch-built Pompe-specific activity (R-PAct) scale. Neuromuscul Disord. 2013;23(3):256–64. 10.1016/j.nmd.2012.10.024 . 23273871 10.1016/j.nmd.2012.10.024

[30] Al-Awadhi AM, Hasan EA, Sharma PN, Haider MZ, Al-Saeid K. Angiotensin-converting enzyme gene polymorphism in patients with psoriatic arthritis. Rheumatol Int. 2007;27(12):1119–23. Epub 2007/04/19. 10.1007/s00296-007-0349-y . 17440728 10.1007/s00296-007-0349-y

[31] Council MR. Aids to examination of the peripheral nervous system. London: Her Majesty’s Stationary Office; 1976.

[32] Beenakker EA, van der Hoeven JH, Fock JM, Maurits NM. Reference values of maximum isometric muscle force obtained in 270 children aged 4–16 years by hand-held dynamometry. Neuromuscul Disord. 2001;11(5):441–6. Epub 2001/06/19. . 11404114 10.1016/s0960-8966(01)00193-6

[33] van der Ploeg RJ, Fidler V, Oosterhuis HJ. Hand-held myometry: reference values. J Neurol Neurosurg Psychiatry. 1991;54(3):244–7. Epub 1991/03/01. . 2030353 10.1136/jnnp.54.3.244 PMC1014394

[34] van Capelle CI, van der Beek NA, de Vries JM, van Doorn PA, Duivenvoorden HJ, Leshner RT, et al The quick motor function test: a new tool to rate clinical severity and motor function in Pompe patients. J Inherit Metab Dis. 2012;35(2):317–23. 10.1007/s10545-011-9388-3 . 21912959 10.1007/s10545-011-9388-3 PMC3278629

[35] A. T. S. Committee on Proficiency Standards for Clinical Pulmonary Function Laboratories. ATS statement: guidelines for the six-minute walk test. Am J Respir Crit Care Med. 2002;166(1):111–7. Epub 2002/07/02. 10.1164/ajrccm.166.1.at1102 . 12091180 10.1164/ajrccm.166.1.at1102

[36] Quanjer PH, Stanojevic S, Cole TJ, Baur X, Hall GL, Culver BH, et al Multi-ethnic reference values for spirometry for the 3-95-yr age range: the global lung function 2012 equations. Eur Respir J. 2012;40(6):1324–43. 10.1183/09031936.00080312 . 22743675 10.1183/09031936.00080312 PMC3786581

[37] Holm S. A Simple Sequentially Rejective Multiple Test Procedure. Scandinavian Journal of Statistics. 1979;6(2):65–70.

[38] Pinheiro J BD, Debroy S, Sarkar D, {nlme}. Linear and Nonlinear Mixed Effects Models. 2015.

[39] A Language and Environment for Statistical Computing [Internet]. R Foundation for Statistical Computing. 2015.

[40] Ausems MG, Verbiest J, Hermans MP, Kroos MA, Beemer FA, Wokke JH, et al Frequency of glycogen storage disease type II in The Netherlands: implications for diagnosis and genetic counselling. Eur J Hum Genet. 1999;7(6):713–6. 10.1038/sj.ejhg.5200367 . 10482961 10.1038/sj.ejhg.5200367

[41] Van Der Ploeg AT, Kruijshaar ME, Toscano A, Laforet P, Angelini C, Lachmann R, et al European recommendations for starting and stopping enzyme replacement therapy in adult patients with Pompe disease: a ten-year experience. Submitted. 2017. 10.1111/ene.13285 28477382

[42] van der Wal E, Bergsma AJ, van Gestel TJM, In ’t Groen SLM, Zaehres H, Arauzo-Bravo MJ, et al GAA Deficiency in Pompe Disease Is Alleviated by Exon Inclusion in iPSC-Derived Skeletal Muscle Cells. Mol Ther Nucleic Acids. 2017;7:101–15. Epub 2017/06/19. 10.1016/j.omtn.2017.03.002 . 28624186 10.1016/j.omtn.2017.03.002 PMC5415960

[43] Tsianos G, Eleftheriou KI, Hawe E, Woolrich L, Watt M, Watt I, et al Performance at altitude and angiotensin I-converting enzyme genotype. Eur J Appl Physiol. 2005;93(5–6):630–3. Epub 2004/12/04. 10.1007/s00421-004-1284-1 . 15578201 10.1007/s00421-004-1284-1

[44] Tsianos G, Sanders J, Dhamrait S, Humphries S, Grant S, Montgomery H. The ACE gene insertion/deletion polymorphism and elite endurance swimming. Eur J Appl Physiol. 2004;92(3):360–2. Epub 2004/05/13. 10.1007/s00421-004-1120-7 . 15138837 10.1007/s00421-004-1120-7

[45] Woods D, Hickman M, Jamshidi Y, Brull D, Vassiliou V, Jones A, et al Elite swimmers and the D allele of the ACE I/D polymorphism. Hum Genet. 2001;108(3):230–2. Epub 2001/05/17. . 11354635 10.1007/s004390100466

[46] Williams AG, Rayson MP, Jubb M, World M, Woods DR, Hayward M, et al The ACE gene and muscle performance. Nature. 2000;403(6770):614 Epub 2000/02/25. 10.1038/35001141 . 10688186 10.1038/35001141

[47] Woods DR, Montgomery HE. Angiotensin-converting enzyme and genetics at high altitude. High Alt Med Biol. 2001;2(2):201–10. Epub 2001/07/10. 10.1089/152702901750265305 . 11443001 10.1089/152702901750265305

[48] Folland J, Leach B, Little T, Hawker K, Myerson S, Montgomery H, et al Angiotensin-converting enzyme genotype affects the response of human skeletal muscle to functional overload. Exp Physiol. 2000;85(5):575–9. Epub 2000/10/20. . 11038409

[49] Vaughan D, Brogioli M, Maier T, White A, Waldron S, Rittweger J, et al The Angiotensin Converting Enzyme Insertion/Deletion Polymorphism Modifies Exercise-Induced Muscle Metabolism. PLoS One. 2016;11(3):e0149046 10.1371/journal.pone.0149046 . 26982073 10.1371/journal.pone.0149046 PMC4794249

